# SOX2 and p53 Expression Control Converges in PI3K/AKT Signaling with Versatile Implications for Stemness and Cancer

**DOI:** 10.3390/ijms21144902

**Published:** 2020-07-11

**Authors:** Thorsten Schaefer, Rebekah Steiner, Claudia Lengerke

**Affiliations:** 1 Stem Cells & Hematopoiesis Laboratory, Department of Biomedicine, University of Basel and University Hospital Basel, CH-4031 Basel, Switzerland; claudia.lengerke@unibas.ch; 2 Immunology Laboratory, Department of Biomedicine, University of Basel and University Hospital Basel, CH-4031 Basel, Switzerland; rebekah.steiner@unibas.ch; 3 Internal Medicine II, University Hospital Tübingen, 72076 Tübingen, Germany

**Keywords:** SOX2, p53, PI3K/AKT signaling, stem cells, pluripotency, genome integrity, DNA maintenance, DNA damage response (DDR), transformation, cancer

## Abstract

Stemness and reprogramming involve transcriptional master regulators that suppress cell differentiation while promoting self-renewal. A distinguished example thereof is SOX2, a high mobility group (HMG)-box transcription factor (TF), whose subcellular localization and turnover regulation in embryonic, induced-pluripotent, and cancer stem cells (ESCs, iPSCs, and CSCs, respectively) is mediated by the PI3K/AKT/SOX2 axis, a stem cell-specific branch of the PI3K/AKT signaling pathway. Further effector functions associated with PI3K/AKT induction include cell cycle progression, cellular (mass) growth, and the suppression of apoptosis. Apoptosis, however, is a central element of DNA damage response (DDR), where it provides a default mechanism for cell clearance when DNA integrity cannot be maintained. A key player in DDR is tumor suppressor p53, which accumulates upon DNA-damage and is counter-balanced by PI3K/AKT enforced turnover. Accordingly, stemness sustaining SOX2 expression and p53-dependent DDR mechanisms show molecular–functional overlap in PI3K/AKT signaling. This constellation proves challenging for stem cells whose genomic integrity is a functional imperative for normative ontogenesis. Unresolved mutations in stem and early progenitor cells may in fact provoke transformation and cancer development. Such mechanisms are also particularly relevant for iPSCs, where genetic changes imposed through somatic cell reprogramming may promote DNA damage. The current review aims to summarize the latest advances in the understanding of PI3K/AKT/SOX2-driven stemness and its intertwined relations to p53-signaling in DDR under conditions of pluripotency, reprogramming, and transformation.

## 1. An Introduction to SOX2 Biology

SRY homology box 2 (SOX2) is a transcriptional modulator imperative to the induction and maintenance of stem cells [1,2]. A given cell qualifies as a “stem cell” if it meets the following functional criteria: stem cells persist in a largely undifferentiated, often dormant physiological default state unless triggered to (1) self-renew and (2) differentiate supporting tissue (re)generation in ontogenesis, homeostasis, and wound healing. This accounts for a wide diversity in stem cell classes, in which SOX2 is a recurrent molecular hallmark.

In healthy conditions, Sox2 (hereafter SOX2, unless a murine origin is otherwise noted) is expressed in ESCs of the morula and the blastula’s inner cell mass (ICM), from which entire organisms can be derived [3]. Deviant SOX2 expression during this developmental stage induces untimely ESC differentiation and is associated with embryonic lethality in mice [4,5]. In later stages of ontogenesis, SOX2 predominantly co-segregates with stem/progenitor cells of the neuro-ectodermal lineage from which epithelial, neuronal, and eye structures derive [6]. Accordingly, several mutant alleles of SOX2 have been identified by their association with micro-/anophthalmology syndromes in humans [7,8], which correlates with Sox2 expression amongst optical cup and retina progenitors in mouse embryos [9,10]. However, SOX2 is also expressed in adult stem/progenitor cells, where it is associated with tissue homeostasis and wound healing (e.g., in murine skin [11]). This significance is also evident in an inducible *Sox2* knock-out model which, upon Sox2 depletion, indicated strong tissue damage and lethality within two weeks [12].

Consistent with the notion that developmental pathways have transforming potential when inadequately or untimely induced, dysregulated SOX2 expression was also reported as a molecular hallmark in human cancer [13,14]. This includes testicular germ cell tumors [15], as well as various carcinomas and gliomas/glioblastomas, that match SOX2’s lineage commitment. In cancer, SOX2 expression frequently coincides with the CSC compartment [16,17,18] from which tumorigenicity, therapy-resistance, and disease relapse are thought to arise [13,19], and moreover with circulating CSC islets as structural correlates of tumor dissemination and metastasis [20,21].

Finally, SOX2 received major attention as a pluripotency inducing transcription factor (TF) in reprogramming technology, where it drives the conversion of terminally differentiated human/murine somatic cells to iPSCs in conjunction with co-factors [2,22]. However, reprogramming can be also imposed by nuclear transfer (i.e., in the absence of ectopic TF expression) [23]. Standardly applied, e.g., in livestock breeding, somatic nuclei carrying a desired phenotypic predisposition can be transferred for reprogramming into de-nucleated oocytes expressing SOX2 [24]. Taken together, various lines of evidence define SOX2 as a critical co-inductor and/or maintenance factor in healthy, diseased, and induced stem cell settings.

## 2. Molecular–Functional Aspects of SOX2-Imposed Stemness

The SOX/Sox family of TFs comprises 20 individual members in mice and humans, of which SOX is the most studied [25]. These proteins share a near invariant DNA binding element, the high mobility group (HMG) [26], with the transcriptional master regulator of virility, SRY [27]. The term SOX (SRY homology box) indicates this descendance. SOX proteins are subclassified by the relative localization of the HMG within their protein sequence and further DNA motifs shared only amongst individual family members [28]. In the derived hereditary tree, SOX2 clusters in the SOX-B1 subfamily that further comprises SOX1 and SOX3. SOX1 and SOX3 can also support reprograming and can substitute for SOX2 in iPSC induction from mouse embryonic fibroblasts (MEFs), although at considerably lower efficacy rates [29]. More distantly related SOX proteins exert distinct biological functions [30,31] and accordingly, do not support iPSC induction [29]. It is noteworthy that while classical reprogramming protocols standardly involve OCT4, KLF4, cMYC, and SOX2 (so-called OKMS reprogramming) [2,22], more advanced procedures have since been described in which individual reprogramming factors can be omitted [32,33], SOX2 can be specifically replaced by TGF-beta inhibitors [34,35], or even the entire array of pluripotency TFs can be surrogated by chemical stimuli [36,37]. However, none of these protocols reached the broad applicability of the OKMS procedure, suggesting the presence of further cell-specific contributions in such non-canonical settings.

Although SOX2 unquestionably interacts with DNA [26,38,39], a direct causal assignment of individual target genes with distinct functional manifestations remains difficult. Indeed, while the human genome comprises several thousand potential docking sites for SOX2, as predicted by an in silico search in advanced human glioma cells [40], an effective association with no more than 489 protein coding genes and 105 pre-miRNAs was experimentally determined by ChIP-seq [40]. A comparable number of bona-fide target factors (699 significantly modulated genes) was also identified by cDNA microarray in nasopharyngeal cancer cells [41]. This target spectrum is further expanded and diversified by interactions with RNA-based polynucleotides [42,43,44]. Accordingly, SOX2’s contributions to stemness are inherently multifactorial and are probably best described as an expression pattern shift.

Given the plethora of individual factors influenced by SOX2, the modulation of its expression inevitably impinges on various physiological features including proliferation, anti-apoptosis, migration, wound healing, epithelial to mesenchymal transition (EMT), clonogenicity in vitro, and elevated tumorigenicity in experimental models in vivo [13,14]. However, investigations in transformed organisms or transformed cells inherently focus on aberrant forms of SOX2 expression and thus potentially give a distorted reflection of SOX2’s true physiological significance. Indeed, in healthy murine ESCs, a modulation of SOX2 expression (either by depletion or overexpression) associates with untimely differentiation phenomena [4,5]. The primordial significance of SOX2 thus is the maintenance of stemness under tightly balanced conditions, while its growth stimulatory significance is context specific and becomes the predominant functional feature in dysregulated settings such as transformation and cancer.

Interestingly, although normally detected in association with DNA in the nucleus, SOX2 actually shuttles between nuclear and cytosolic compartments by virtue of nuclear localization and nuclear export sequences (NLS, NES, respectively), as documented for both human and mouse-derived cell samples [45,46]. This shuttling activity becomes most evident upon AKT pathway inhibition when SOX2 is retained in the cytosol and (if treatment persists) is successively cleared by proteasomal decay [47,48,49,50]. This implies that cells may adaptively tailor their stem cell status by modulation of SOX2’s subcellular distribution in dependence of AKT activity. Supporting this notion, SOX2 localizes to the nucleus of ESCs (from which the embryo derives as a generative function), while it localizes to the cytosol of trophectoderm cells (that contribute to placenta formation as a vegetative function) in developing mouse embryos [5]. Taken together, SOX2 is a highly promiscuous and versatile transcriptional modulator that imposes expression pattern shifts critical for stemness induction and maintenance, although its significance may not be confined to merely these roles.

## 3. The PI3K/AKT/SOX2 Axis in Stemness, Reprogramming, and Cancer

**Figure 1:**
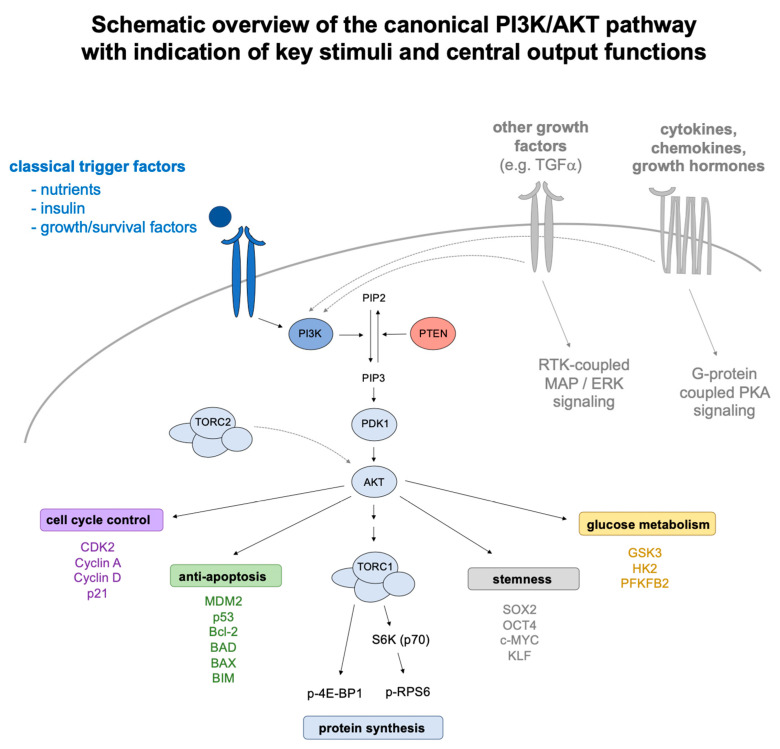
Schematic overview of the canonical PI3K/AKT pathway with indication of key stimuli and central output functions. The PI3K/AKT signaling pathway is a versatile molecular module that converts external stimuli into downstream effector functions within the cell. Key trigger factors comprise metabolic cues (such as individual amino acids or insulin) as well as classical growth and survival factors (e.g. FGF, IGF, and others) that dock to cognate receptors on the plasma membrane and induce the conversion of phosphatidyl-inositol (4,5,)-bisphosphate (PIP2) into phosphatidyl-inositol (3,4,5,)-trisphosphate (PIP3), which acts as second messenger in the pathway. This conversion is catalyzed by enzymes of the PI3 kinase (PI3K) family, which comprises several distinct subclasses and isoforms expressed in a tissue-selective, yet overlapping manner. PIP3 recruits and activates PDK1 at the plasma membrane, which in turn activates AKT, a Ser/Thr kinase also known as protein kinase B (PKB). AKT serves as a central relay factor in the pathway from which glucose metabolic, anti-apoptotic, cell-cycle stimulatory, and stemness-sustaining activities arise (here illustrated in different color codes, with further indication of selected downstream factors). One downstream scaffold of exceptional significance has been identified in mTORC1, a multi-factorial protein complex that chiefly regulates cellular protein synthesis via the phosphorylation of the translation co-regulator 4E-BP1 and the phosphorylation target of S6-kinase (S6K), the ribosomal small subunit component RPS6. The pathway is counter-balanced by PTEN, a phosphatase implicated in the turnover of PIP3. Further regulatory input is provided through crosstalk with related growth stimulatory modules such as MAP/ERK or PKA-signaling pathways that respond to other growth factors, cytokines, chemokines, or hormones. It is noteworthy that basic PI3K/AKT signaling is also maintained in the effective absence of external stimuli by related cellular relays (e.g., mTORC2) and auto-regulatory feedback mechanisms that warrant the expression of housekeeping factors under conditions of cellular dormancy or senescence. Finally, the PI3K/AKT axis also attains particular relevance in transformation and cancer, where hot spot mutations in the genes encoding for PTEN, PI3K, and AKT enforce constitutive forms of pathway activation with high prevalence.

Proliferation and differentiation depend on cell cycle progression and adjusted cell mass growth (i.e., aggravated biosynthetic processes) as underlying molecular principles. While numerous individual factors impinge on these mechanisms, the PI3K/AKT signaling axis resembles a primordial functional module of superordinate significance for mass growth and adjusted proliferation control [51]. In its key components, conserved from unicellular eukaryotic organisms to higher vertebrates and humans, the PI3K/AKT pathway converts nutrient and growth factor sensing on the plasma membrane into downstream effector functions within the cell [52]. Central metabolic stimuli, including glutamate as an indicator of amino acid availability [53] and insulin as a surrogate marker of sugar [54], trigger TORC1-dependent protein synthesis as a fundamentum of mass growth [53,55,56], and cyclin kinase-driven cell cycle progression as a pacemaker of adjusted proliferation [57,58]. For a more comprehensive overview, please refer to Figure 1, which illustrates external trigger factors, key signaling components, and central output relays jointly understood as manifestations of PI3K/AKT signaling.

In dormant stem cells, where growth and proliferation are largely dispensable or even effectively undesired, PI3K/AKT signaling may be broadly attenuated so that somatic stem cells are, on average, smaller (with cell size being an indicator of protein synthesis, see above) and show an overall decreased translation rate when compared to surrounding non-stem cells [59,60]. In stem cells actively involved in tissue regeneration, growth and proliferation become highly desirable functional features instead. For example, in tissue replenishing stem cells, FGF/EGF-triggered PI3K/AKT signaling critically contributes to wound healing [61]. In cancer, where constitutive PI3K/AKT signaling has long been identified as a recurrent driver axis [62,63], a more specific stem cell involvement can be deduced, e.g., from *PIK3CA(H1047R)* induced trans-conversion of luminal and basal cell layers of the mammary gland in mouse from which composed, multi-lineage carcinomas derive [64]. Accordingly, PI3K/AKT signaling is a critical determinant of stem cells and adaptively tailored to local demand, whereas dysregulated PI3K/AKT signaling is more specifically associated with cancer and stem cell-driven tumorigenesis.

In line with these findings, we and others identified the PI3K/AKT axis as a central mediator of SOX2’s subcellular distribution and turnover regulation in ESCs, iPSCs, and CSCs of mouse and human [65]. A first indicator of such connectivity was the PI3K/AKT cross-reactive inhibitor perifosine, which attenuated PTEN-mutant induced outgrowth of human mammary epithelium-derived stem cells [66]. Subsequent analyses reconfirmed this observation and further dissected the roles of PI3K and AKT as a knock-down of AKT expression alone impaired clonogenicity arising from breast carcinoma stem cells [67]. A molecular–functional relation to SOX2 in this context was first established by studies in mouse, where the Akt-imposed phosphorylation of Sox2 was linked to ESC maintenance [68] and iPSC induction [69]. Further underscored by findings in human cancer cells, PI3K/AKT signaling sustains nuclear entry and DNA modulatory functions of SOX2, whereas in AKT-inhibited cells, SOX2 is retained in the cytosol and is successively cleared by proteasomal turnover [47,48,49,50]. Suggesting a potential applicability of these findings in the fight against cancer, AKT inhibition attenuates clonogenicity effects of human breast cancer-derived cells in vitro [47] and tumorigenicity from nasopharyngeal cancer cells upon xenotransplantation in vivo [48]. Furthermore, while cytostatic drugs standardly applied in the treatment of breast cancer (e.g., cisplatin or paclitaxel) impose a selection advantage for CSCs, AKT inhibition impairs proliferation of CSCs and bulk tumor cells alike [47]. These observations have since been confirmed in various cell types and cancers, as recently summarized in a review on SOX2 secondary modifications in stemness, reprogramming, and cancer [65].

## 4. DNA Damage Control in Stemness and Reprogramming

The maintenance of genome integrity is a functional prerequisite for stem cells, from which organs or even entire organisms derive and where the repopulating potential is passed on from one generation to the other. A cell’s genome, however, is constantly challenged by environmental risk factors and cell-intrinsic influences that impose damage to DNA [70]. If not stringently repaired, these mutations successively accumulate and increase the risk of transformation [71]. Long-lived cell types (such as quiescent stem cells) or clonal lineages derived from stem cell founders, are particularly affected. However, an array of intertwined molecular relays (collectively referred to as DNA damage response—DDR) has evolved to correct for the various different forms of DNA damage that arise [72,73]. Mutations in these repair factors not only associate with an elevated incidence of cancer [74,75,76], but also with progeria syndromes where there is compelling evidence for stem cell-specific contributions [77,78,79,80]. 

This interplay of aging and stemness is unquestionably best resolved in the hematopoietic system, which is described here as an archetype of tissue-replenishing stemness. The blood system is qualitatively maintained by stem cells (hematopoietic stem cells, HSCs), but quantitatively replenished by their derived progenitor populations (multi-potent progenitors, MPPs), which still retain full lineage differentiation potential but no longer possess self-renewal capacity, and further, more lineage-restricted species. In many experimental settings, these populations are not strictly discriminated and thus often jointly referred to as hematopoietic stem/progenitor cells (HSPCs) or else, defined as long-term vs. short-term HSCs, respectively. However, only individual HSCs transiently cycle to replenish the progenitor pool, whereas the major part of the murine HSC compartment persists in G_0_ dormancy [81,82], and thus is not surveyed by cell cycle-checkpoints and their associated DNA integrity control mechanisms. DNA damage accumulating in quiescent murine HSCs is, however, largely resolved as individual HSCs re-enter the cell cycle [83]. Leukemia, by contrast, arises from consecutive mutational hits within divisionally active HSPCs and their derived clonal lineages [84]. Nonetheless, the incidence of neoplastic conversions gradually increases with age and aged HSCs show severe functional impairments over juvenile counterparts with regards to homing, engraftment, lineage reconstitution, and self-renewal capacities, as deduced from blood reconstitution analyses in mice [85]. However, such age-dependent comparisons are inherently confounded by overlapping epigenetic changes and thus not exclusively linked to mutational burden [86]. The specific significance of DDR mechanisms for genome integrity in HSCs is therefore better indicated by mouse models, where distinct DDR mutations functionally associate with impaired hematopoiesis under respective stress settings [87,88,89]. In line with these murine studies, human HSCs are particularly sensitive to irradiation damage, characterized by delayed double-strand break repair (DSBR), by persisting, non-resolved DNA foci, and by aggravated apoptosis over equally irradiated progenitor populations [90]. These data further emphasize that in quiescent HSCs, DNA repair mechanism are overall less active and damaged HSCs are preferentially sacrificed to maintain a pristine stem cell pool. 

Specific adaptations in the DNA control mechanisms of mESCs were reported as early as 1993, when their in vitro differentiation was noted to coincide with an increasing risk of UV-imposed DNA damage [91]. Subsequent comparative analyses with littermate somatic cell types confirmed these results and revealed enforced embryonic resilience to further mutagens such as oxidative stress [92]. DDR mechanisms are thus generally enforced in actively cycling mESCs, as also confirmed for human ESCs, where cDNA arrays indicated aggravated expression of DDR genes and comet assays illustrated improved DNA integrity over somatic cells when exposed to various individual mutagens (H_2_0_2_, UV, or psoralen) [93]. Conversely, defects in mismatch repair, nucleotide excision repair, and non-homologous end joining (MMR, NER, and NHEJ, respectively) associated with impaired stem cell functions and accelerated murine stem cell exhaustion [94,95]. However, as already seen for HSCs, a pristine stem cell population may not only be maintained through repair mechanisms, but also by the elimination of aberrant clones. In line with this notion, irradiation and further stress factors have been associated with facilitated apoptosis amongst mESCs as well [96,97]. Accordingly, while ESC may also be further sub-classified into distinct pluripotency states (naïve, primed, and ground state [98]), they are on average privileged over somatic cells and respond to DNA damage in more efficient ways (i.e., by aggravated repair or else, facilitated apoptosis). In total, these differences account for an approximate 100-fold lower mutational burden in mESCs than in isogenic MEFs or adult somatic tissue cells (10E-4 vs. 10E-6, respectively) [99,100].

iPSCs resemble natural stem cells in several physiological characteristics, including DNA maintenance mechanisms such as the reduction of mitochondria to minimize DNA damage arising from reactive oxygen species (ROS) [101]. Although shared pluripotency characteristics identify iPSCs as a functional homologue of ECSs, the expression profiles of these cell types do not perfectly match [102]. This distinction is particularly evident on the mutational level as well. While the mutational burden of healthy ESCs is markedly lower than in their derived somatic populations (see above), the mutational load of human iPSCs exceeds that of their somatic cell origin and only declines gradually thereafter with extended cultivation time [103]. Such reprogramming induced hypermutation was also reported for murine iPSCs, irrespective of whether pluripotency was induced by integrative or non-integrative viral transformation, although the latter is generally considered the “safer” procedure [104]. Accordingly, while the integration of viral particles clearly imposes an additional risk factor, the immanent genetic reorganizations that underlie de-differentiation and pluripotency induction may evoke DNA damage *per se* [103,104]. This technology-immanent elevated risk of DNA damage provides one potential explanation as to why reprogramming efficacy, despite serious improvement throughout the past years, remains modest overall (i.e., while unfit iPSCs are statistically eliminated through clonal selection, more compatible but nevertheless mutated clones may be eliminated through apoptosis). Accordingly, mutations in cell cycle regulators and apoptosis inductors (such as p21 and p53, respectively) raise reprogramming efficacy and accelerate the induction rates of human/murine iPSCs, but concomitantly increase the risk of genetic aberrations [105,106,107,108,109]. Conversely, mutations acquired though reprogramming often hit tumor suppressor genes or DDR factors [110]. Jointly, these system immanent liabilities remain major obstacles for an otherwise highly desirable applicability of iPSCs in homologous regenerative medicine.

Taken together, the extent and regulation of DDR mechanisms clearly distinguishes stem cells from somatic cells, while individual stem cell populations follow distinct DNA maintenance strategies, as exemplified here for HSCs, ESCs, and iPSCs.

## 5. AKT/p53 Antagonisms Balance Cell Cycle Progression and DNA Damage Control

The complexity of DDR modules, their dynamics, and the adaptive changes in response to external stimuli, are potentially best illustrated by integrative system biology approaches [111]. While such models incorporate numerous individual elements, they converge on a number of common nodes that define single factorial contributions of superordinate significance. One such relay factor is the tumor suppressor p53 (historically TP53, gene name *TP53*, hereafter consequently p53), a multi-functional protein that, among other activities, associates with DNA to impose transcription modulatory effects [112,113]. Underscored by phylogenetic conservation, a primordial function of p53 lies in DNA damage-imposed cell cycle arrest (to provide time for repair mechanisms) and subsequent, conditional apoptosis (selectively in those cells, whose DNA integrity cannot be maintained or restored) [114]. This sequence of events correlates with a gradual increase of p53 protein abundance in DNA damaged cells [115] and, furthermore, with overall higher affinities to the DNA recognition elements of cell cycle regulatory, rather than apoptosis inductory genes [116]. 

p53’s anti-proliferative, pro-apoptotic roles in response to (replication) stress inherently antagonize with growth stimulatory, anti-apoptotic signaling pathways such as the PI3K/AKT axis that triggers the cell cycle in dependence of trophic stimuli and growth factor sensing (see preceding paragraphs). These evidently opposing roles are balanced by intertwined, antagonist regulatory mechanisms [117]. In particular, while AKT-imposed phosphorylation enforces nuclear entry of MDM2, an E3-type ubiquitin ligase that promotes proteasomal degradation of p53 [118], p53 induced caspase cascades enforce degradation of both MDM2 and AKT [119]. This immediate cross-talk is augmented by further accessory relays, such as the AKT-imposed, phosphorylation-dependent inhibition of pro-apoptotic Bad [120] or p53-driven, enforced transcription of PTEN as a natural antagonist of PI3K/AKT signaling [121]. However, p53 expression is largely compromised unless MDM2 becomes inactivated, mostly via stress-imposed phosphorylation ARF [122]. Accordingly, the here outlined antagonism between p53 and AKT activities may only prevail under conditions of effective DNA stress (i.e., when the MDM2-imposed turnover control of p53 is overcome). 

By contrast, in the absence of DNA damage, AKT sustains p53 baseline expression via mTORC1-mediated translational control and, in this context, correlates with p53-imposed cellular senescence, not apoptosis [123]. In cancer, where regulatory networks are often distorted and physiological settings perturbed, mutant forms of p53 display gain-of-function phenotypes and accumulate to degrees not effectively seen for wild-type p53 [124,125]. This specifically includes cells with constitutive PI3K/AKT signaling, so that also in transformed settings, AKT cannot be considered a stringent antagonist of p53 expression [126]. 

**Figure 2:**
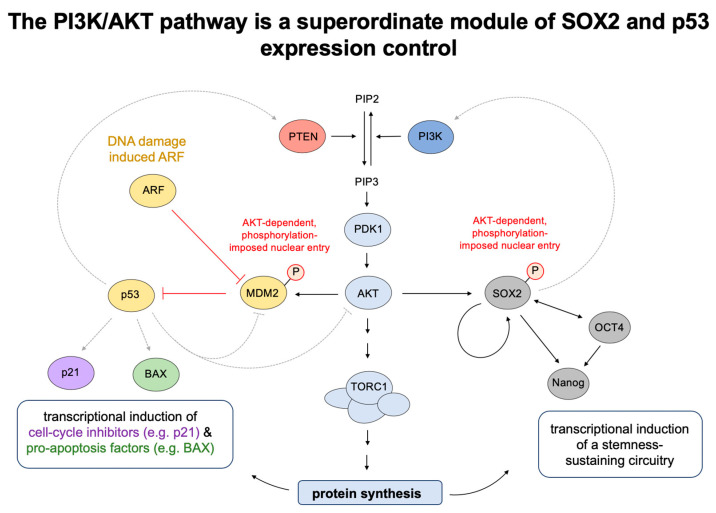
The PI3K/AKT pathway is a superordinate module of SOX2 and p53 expression control. The PI3K/AKT pathway (blue) resembles a superordinate regulatory module for the expression control of SOX2 (grey), a stemness inducing transcriptional master regulator (see chapter 3), and the tumor-suppressor p53, a central transcriptional modulator at the intersection of DNA-damage control (yellow), apoptosis induction (green), and cell-cycle progression (purple). Note an overt degree of molecular symmetry between these output functions, which shows in (1) AKT-enforced nuclear entry of key components (i.e., MDM2 and SOX2), (2) corresponding transcriptional adaptations that modulate the expression of respective downstream effectors, (3) a mutual dependence on PI3K/AKT-regulated protein synthesis to translationally impose these expression changes, and finally (4) near-symmetric feedback regulatory relays to balance and adjust these output functions. These interrelations are of particular significance for stem cells, whose genome integrity is a functional imperative. In cancer, though, the indicated circuitry is frequently distorted by constitutively activating mutations in PI3K/AKT signaling and/or functional disruptions within DNA response elements that rank amongst the most prevalent transforming aberrations.

Taken together, while AKT counteracts an accumulation of p53 in the absence of DNA damage, it sustains p53 baseline expression via TORC1, whereas analyses in p53^mutant^ conditions indicate further regulatory relays that gain particular significance when p53’s structural integrity and canonical effector functions are distorted [127]. These basic concepts of p53 expression control are illustrated in Figure 2, with further indication of PI3K/AKT/SOX2-imposed stemness as an overlapping molecular module. 

## 6. P53 Mutant-Dependent, Impaired Apoptosis and PI3K/AKT Enforced Growth Signaling Synergize in Cancer

Due to its inherently anti-proliferative, mutation-resolving significance, a functional disruption of p53 signaling ranks amongst the most prevalent mutations in cancer [128]. However, mutations in p53 have comparatively little transforming potential on their own, may not significantly raise tumorigenesis unless triggered by external stressors (UV, IR, cytostatic drugs), or depend on additional driver mutations to boast clonal expansion. By contrast, mutations in proliferation drivers (such as Ras) may initially boast the cell cycle, but the concomitant accumulation of DNA damage rapidly forces cells into senescence thereafter. This has been deduced, for example, from Ras mutant human fibroblast cell lines xenotransplanted into immunocompromised mice [129]. Interestingly, under conditions of constitutive PI3K/AKT signaling (either induced by depletion of *PTEN*, expression of constitutively active *PI3KCA* alleles, or mutations in *AKT1*), cellular senescence can be imposed among human fibroblast cells even without accumulation of DNA damage [123]. These mechanisms involve the enforced TORC1-imposed translation of p53 protein and phosphorylation-based inhibition of its turnover regulator, MDM2 [123]. Accordingly, although PI3K/AKT signaling and p53 control mechanisms mostly antagonize, the disruption of either one axis generally is insufficient to induce cancerous transformation.

Remarkably, although a cooperative distortion of proliferation control and counteracting apoptosis regulation seemingly matches the “hallmarks of cancer” paradigm [130], it is considerably less certain if deviant PI3K/AKT signaling and p53 dysregulation functionally synergize in patient settings or merely co-exist and overlap due to individually high incidence rates. Whereas functional synergism with cooperatively impaired prognosis has been reported for bladder [131,132] and endometrial cancers [133], no evidence for such cooperativity was found in head and neck squamous cell carcinoma [134] or eosophagial cancers [135], despite high coincidence rates. In mammary carcinoma, exon 20 mutations in *PIK3CA* were described to correlate with *TP53* mutations in one study [136], whereas mutant *TP53* preferentially associates with triple negative BC, and *PI3KCA* mutations predominantly associate with ER + /PR+ BC in another [137]. Accordingly, while the functional synergy between dysregulated PI3K/AKT and p53 relays may seem self-explanatory, in clinical practice, such cooperativity remains case-dependent or reaches statistical significance only in certain cancer entities or patient subsets.

This inconsistency may be largely explained by differences between effective p53^null^ settings and functionally impaired, but nevertheless expressed, p53^mutant^ alleles [138]. In fact, while p53 expression is largely compromised by mutations in upstream *ARF*, the *TP53* locus itself is rarely deleted in cancer and nearly all naturally occurring p53 mutants have a spontaneous, somatic origin (except for some rare hereditary forms, e.g., in Li-Fraumeni-syndrome) [127]. Moreover, most cancer patient-derived p53 mutants are missense mutations that predominantly concern a small number of mutational hot spots, with >70 percent of all polymorphisms affecting the protein’s DNA binding domain [139]. Accordingly, while p53 single residue mutants may be impaired in DNA binding (and thus lose their pro-apoptotic, tumor suppressive transcriptional role) [124,125] various mutant forms of p53 are stably expressed and effectively accumulate to degrees not equally seen for the wild-type form of the protein [126,140]. Some mutants even elicit gain-of-function effects, supposedly reflecting perturbations in other DDR relays that selectively manifest in p53^mut^ conditions [138]. Tumorigenicity arising from p53^(R172H)^ mutant mice, for example, repeatedly tested higher than that of p53^null^ littermates [141]. Individual mutations prove particularly detrimental under conditions of enforced DNA stress, such that classical cytostatic drug treatments or reduction therapies (involving paclitaxel, doxorubicin, or γ-irradiation) may paradoxically impose adverse effects, as can be deduced, e.g., from p53 mutant-dependent responses to neoadjuvant therapy in breast cancer [141,142].

These notions may help explain some of the historic inconsistencies that relate to the prognostic significance of p53 in cancer. For example, in mammary carcinoma p53 mutations have been associated with impaired clinical outcome in primary patients [143], whereas the histopathological detection of p53 expression by antibody staining was associated with beneficial outcomes selectively in *HER2^+^* breast cancer [144]. In light of the above notions, the significance of such analyses (and of the cooperativity studies mentioned further above) is strongly compromised unless distinct p53 alleles are described in consideration of individual patient history (i.e., treatment-imposed DNA stress cycles and concomitant stages of clonal selection) and further reference to *ARF* status.

In selected cancers, p53 and AKT signaling may functionally synergize without underlying mutational distortions. Indeed, while most tumors overexpress telomerase for immortalization, app. 15% of cancers maintain functional telomeres by mechanisms jointly referred to as alternative lengthening of telomeres (ALT) [145]. These involve homologous recombination events that often induce (sub)telomeric lesions as indicated by telomer dysfunction-induced loci (TIFs). TIF scoring indicated inherently higher DNA damage rates in ALT cancer cell lines (VA-13, U2OS, SAOS2, and SKLU-1) than in telomerase-positive, non-ALT cancer cells (MCF7, A549), or healthy-human BJ-fibroblasts. These findings correspond with slightly increased p53 levels in ALT over non-ALT references [146]. Such residual p53 expression may be insufficient to stall the cell cycle and trigger the expression of apoptosis inductors but it has been proposed to transcriptionally activate Rictor and, consequently, TORC2-induced AKT-driven proliferation of ALT tumor cells [146]. Accordingly, the functional significance of wild-type p53 remains case dependent and its expression is often paradoxically maintained in cancer. Furthermore, wild-type p53 has been described to suppress ferroptosis (iron-induced cell death) in cysteine deprived human cancer cell lines of variable tissue origin [147], and to act as a driver of tumorigenesis specifically in hepatocellular carcinoma where it contributes to the attenuation of oxidative phosphorylation [148]. Jointly, these findings indicate that endogenous p53 and AKT activities may not inevitably antagonize, but occasionally also synergize in cancer within certain concentration windows or individual disease settings.

Advanced tumor stages and relapsing cancer(s) are often characterized by treatment-imposed clonal selection effects that favor the development of a resistance mechanism. Specifically in epithelial and neuronal cancers, these effects often coincide with SOX2 expression [16,17,18,19] and aggravated PI3K/AKT signaling as a superordinate module of SOX2 post-transcriptional stabilization [65]. However, while treatment-imposed DNA damage may either induce or favor mutations that help overcome DDR mechanisms (e.g., by disruption of *TP53* or *ARF*), other cancers are paradoxically characterized by aggravated DDR. This involves, for example, IR-resistant forms of hepatocellular carcinoma. In this indication, the appearance of poorly differentiated, stem cell like features coincides with enforced AKT signaling, a joint induction of SOX2 and p53 mRNAs, and enhanced repair mechanisms to resolve IR-imposed DNA lesions [149]. Taken together, the interplay of PI3K/AKT and p53 in cancer presents highly divergent. While constitutive PI3K/AKT signaling and impaired, p53-dependent DDR relays may synergize in various cancers, their endogenous forms have also been described to potentially cooperate in selected disease types, tumor stages, or treatment conditions.

## 7. PI3K/AKT/SOX2 Signaling and p53-Dependent Apoptosis Regulation in Stem Cells

Modulations in p53 expression are not only of relevance to cancer (stem) cells, but they also impact iPSCs induction. In fact, a depletion of p53 strongly increases reprogramming efficacy by suppression of apoptosis, but at the expense of DNA integrity [150]. Conversely, in other stem cell settings, a depletion of p53 has been associated with improved DNA quality. For example, in HSPCs derived from Fanconi anemia patients (an inherited DNA repair deficiency syndrome characterized by progressive bone marrow failure with juvenile onset), p53 is endogenously hyperactivated due to replication stress and unresolved DNA lesions [151]. Here, the knock-down of p53 improves DNA quality as HSPCs are no longer forced into apoptosis and eliminated, but rather may re-enter the cell cycle and thus benefit from its associated DNA control and repair systems [152]. Accordingly, the functional significance of p53 in stem cell settings remains case-dependent.

These versatile roles of p53 become particularly evident in systemic conditions in vivo, where individual stem cell compartments exert distinct ontological functions at different times. The embryonic lethality of *Mdm2^−/−^* knock-out mice, for example, is functionally overcome by *Mdm2^−/−^ Tp53^−/−^* co-depletion. The double knock-outs grow into adulthood, but die preterm (at app. month eight) due to tumor burden [153]. Mutant alleles of *TP53*, such as the p53^515C^ hypomorph, likewise restore viability although *Mdm2^−/−^ Tp53^515C/515C^* mice do not reach maturity. These animals succumb to postnatal bone marrow failure, characterized by ROS-induced cell cycle arrest and apoptosis in the hematopoietic compartment [154]. Accordingly, individual stem cell populations are differently challenged by p53, with embryonic lethality linked to dysregulated p53 expression in ESCs, juvenile bone marrow failure arising from p53 mutations in HSPCs, and adult tumorigenesis from p53 deletion in more mature stem/progenitor populations.

In fact, p53 expression may be largely incompatible with the maintenance of stemness. This is evident in the inherently unresolvable molecular antagonism between PI3K/AKT/SOX2-imposed stemness and PI3K/AKT/MDM2-mediated p53 suppression, but also in additional aspects. For example, in DNA-damaged murine ESCs p53 not only associates with response elements that regulate DDR genes, but also with the promoter of *Nanog*, a transcriptional co-inductor of stemness. Indeed, Nanog functionally cooperates with SOX2 not only in ESCs maintenance [155], but also in germ cell-derived tumors [15], in various carcinomas and gliomas [156], and in reprogramming settings [32]. A DNA damage-imposed, p53-enforced transcriptional suppression of Nanog may thus coincide with an effective loss of stemness, as experimentally validated by an induction of differentiation amongst mESCs [157].

Further critical influences arise from the cyclin-dependent kinase inhibitor, p21. While p21 is well known to arrest the cell cycle in response to DNA damage and p53 activation [158], its knock-down is functionally associated with an early exhaustion of HSCs and neuronal stem cells (NSCs) in mice [159,160]. In NSCs specifically, p21 has been implicated in the attenuation of a *Sox2* enhancer element, SRR2 [161], which otherwise sustains Sox2 expression through autocrine self-induction (e.g., imposed by Sox2/Oct4 complexes in mESCs) [162]. Hence, next to the aforementioned feedback regulation of Nanog, the cell-cycle regulator p21 also offers a regulatory relay for stemness control through expression modulation of SOX2. Conversely, SOX2 overexpression in p21 knock-down cells enforces the DNA damage characteristic of replication stress and a corresponding induction of p53 [161]. Accordingly, p21, p53, and SOX2 create a functional triangle that balances cell cycle control, apoptosis induction, and stemness in stem cell populations such as NSCs.

Finally, various further cell cycle regulators and apoptosis factors besides the here featured p21, p53, and MDM2 proteins underly adaptive modulation through PI3K/AKT-imposed phosphorylation (e.g., BAD, BRCA1, CASP9, to name a view) and further downstream mTORC1-associated relay functions, which vastly exceed mere protein expression control [163]. Accordingly, the PI3K/AKT axis resembles a superordinate module of SOX2-imposed stemness and p53-dependent apoptosis control, while various additional contributions impinge on this balance and offer regulatory options for case-dependent fine tuning, as exemplified here for Nanog and p21.

## 8. Concluding Remarks and Outlook

Research over the past few years characterizes SOX2 as (1) a transcriptional regulator of stemness in early embryogenesis and in ontologically more advanced, mostly neuro-ectodermal stem/progenitor cells, (2) a devastating oncogene in germ line-derived tumors, neuronal cancers and various carcinomas, and (3) a critical co-inductor in the reprogramming of terminally differentiated somatic cells to iPSCs. A unifying molecular module in all these settings has been identified as the PI3K/AKT/SOX2 axis, a stem cell-specific branch from the canonical PI3K/AKT pathway that mediates SOX2 nuclear entry and turnover. The PI3K/AKT/SOX2 axis thus provides an attractive lever to improve reprogramming efficacy and to address some of the safety concerns associated with iPSC induction. At the same time, this axis may offer a molecular mechanism for therapeutic intervention in *SOX2^+^* cancers and, more specifically, against *SOX2^+^* CSCs.

The link to PI3K/AKT signaling and its growth promoting, anti-apoptotic significance seemingly matches the functional requirements of divisionally active stem cells (e.g., in early ontogenesis or wound healing). A constitutive activation of PI3K/AKT signaling is also frequently seen in cancer, where SOX2 expression functionally coincides with the CSC compartment and its implication in treatment resistance, tumor dissemination, and relapse. Here, the PI3K/AKT/SOX2 axis may be augmented by further distortions in DNA control and apoptosis relays (mostly by mutations in either *TP53* and/or *ARF*) which establishes functional synergy in some, but not all settings.

Although advantageous under proliferative conditions, the immanent link to PI3K/AKT is detrimental to stem cell dormancy when cell cycle progression must be suppressed and protein synthesis (as a driving force of differentiation) is attenuated to a basic maintenance level. Similarly, PI3K/AKT-associated anti-apoptosis is a relevant challenge for germ cells and ESCs, where genomic integrity must be maintained despite high proliferative capacity. In iPSCs, the balance between proliferation control and DNA maintenance is severely perturbed. However, whereas transient stem cell conditions and trans-differentiation events are natural phenomena, exogenously enforced somatic cell reprogramming remains an artificial intervention.

**Figure 3:**
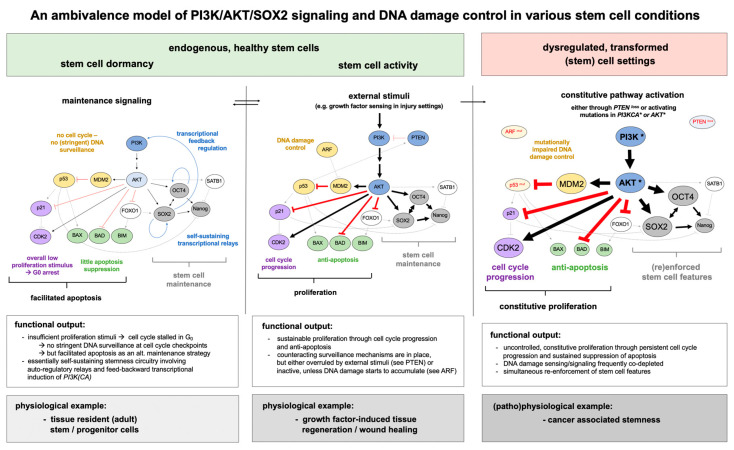
An ambivalence model of PI3K/AKT/SOX2 signaling and DNA damage control in various stem cell conditions. The PI3K/AKT signaling pathway is a versatile molecular module that branches into intertwined downstream relays, each of them linked to cognate cell biological outputs such as cell proliferation, anti-apoptosis, and stem cell maintenance. For comprehensiveness, each relay is represented by a select number of key constituents and critical intermediary nodes. In particular, the pluripotency factors SOX2, OCT4, and Nanog are illustrated as mediators of stemness (grey), BAX, BAD, and BIM as examples of apoptosis regulators (green), p21 and CDK2 for cell cycle control (purple), and ARF, MDM2, and p53 as an intermediate relay linked to DNA damage control (yellow). The interplay of these modules is adaptively tailored to physiological requirements and thus varies between individual stem cell conditions. Stem cell dormancy (left) is primarily characterized by self-sustained maintenance signaling and an overall cell cycle arrest that compromises stringent DNA surveillance but facilitates apoptosis as an alternative maintenance strategy for a pristine stem cell pool. Proliferative stem cell settings (center), are characterized by synergist functional effects between PI3K/AKT sustained stemness, cell cycle progression, and anti-apoptosis, that are balanced by counteracting relays (i.e., DNA damage control or pathway inactivation by PTEN). In dysregulated stem cell conditions, e.g., upon transformation (right), the PI3K/AKT axis is often mutationally distorted so that growth and stemness are constitutively enforced. These effects are further exacerbated in cancers where DDR components (mostly p53 and/or ARF) are co-mutated. Here, proliferation is largely uncoupled from counterbalancing control mechanisms.

Accordingly, the interplay between PI3K/AKT/SOX2-imposed stemness and more canonical PI3K/AKT effects is challenging for stem cells, requiring compromises and individual stem cell compartments to follow distinct strategies. A successive accumulation of DNA damage, for example, may be tolerated in dormant stem cells (such as HSCs) and is only secondarily cleared as individual cells re-enter the cell cycle. Other compartments (e.g., ESCs) follow the opposite strategy and enforce apoptotic mechanisms to warrant stem cell integrity. Overall, the interplay between stem cell induction and maintenance, and DNA control and repair does not converge in a unifying concept, but must be adaptively tailored to distinct stem cell requirements (see Figure 3 for exemplification).

Stemness and DNA control mechanisms also attain particular significance in cancer, as can be deduced from the individually high incidence rates and occasionally synergistic mutational distortion of *PTEN/PIK3CA/AKT1* and *ARF/TP53* in tumors. Here, PI3K/AKT-sustained SOX2 expression and dysfunctional DDR specifically relate to the development of resistance effects, to CSC dormancy despite clinical remission, and often to unmet medical need particularly in relapsing disease. Accordingly, these dynamics functionally contribute to the major obstacles for a sustained, long-term cure to cancer. The highly diversified examples of stemness-related DNA maintenance strategies summarized in this article argue that these hurdles will unlikely be overcome by comprehensive approaches, while hope for future advances lies within personalized strategies.
